# 1068. MicroRNA-regulated immunosuppression in severely injured polytrauma patients

**DOI:** 10.1186/2197-425X-2-S1-P84

**Published:** 2014-09-26

**Authors:** HC Owen, HD Torrance, K Brohi, CJ Hinds, MJ O'Dwyer

**Affiliations:** Centre for Translational Medicine and Therapeutics, William Harvey Research Institute, Barts and the London School of Medicine and Dentistry, London, UK; Adult Critical Care Unit, Royal London Hospital, Barts Health NHS Trust, London, UK; Centre for Trauma Sciences, Blizard Institute, Barts and the London School of Medicine and Dentistry, London, UK

## Introduction

We have demonstrated that traumatic injury is associated with an early immunosuppressive response, the extent of which is associated with an increased risk of developing nosocomial infections. In particular, the expression of the immunosuppressive cytokine IL-10 was up-regulated 2 hours following injury [1]. However, the mechanisms involved are unclear. MicroRNAs (miRs) are short non-coding RNA molecules whose main function is to down-regulate gene expression. Although preliminary laboratory data suggest alterations in miR expression may play a role in triggering this immunosuppressive phenotype there is currently a lack of data in trauma patients [2, 3].

## Objectives

To explore alterations in candidate miR expression that may regulate the immunosuppressive phenotype following severe trauma and evaluate their potential role in enhancing the risk of nosocomial infections.

## Methods

Following ethics approval and consent, 30 ICU patients admitted following severe traumatic injury and 16 healthy age and sex matched controls were recruited. miRs were isolated utilising PAX Gene and miRNA-Easy extraction kits (Qiagen). miRs were selected for analysis based on miRBase target prediction scores for the IL10 promoter. Candidate miRs were quantified by qPCR at 2 hours and 24 hours following injury and then normalised to the small nucleolar RNAs RNU44 and RNU48. Infections were assessed using predefined criteria.

## Results

**Figure 1:**
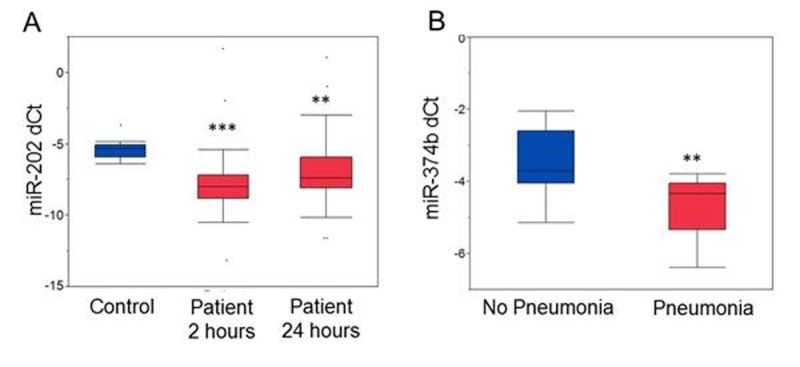
(A) miR-202 expression levels are decreased in patients vs. healthy controls at 2 and 24 hours. (B) miR-374b expression is significantly reduced in patients with pneumonia at 2 hours (**p<0.01; ***p(0.001; median and interquartline ranges)

miR-202, miR-374b and miR-125a3p were selected for analysis as they were in the top 10% of miRs predicted to target the IL-10 promoter. After 2 hours, expression of miR-202 was significantly reduced (38.8%; p< 0.001; Figure 1A) in patients compared to healthy controls. This reduction was maintained (22.1%, p = 0.006) 24 hours after injury, and was associated with the extent of shock (pH (p = 0.009); lactate (p = 0.012) and base excess (p = 0.039)) at admission. miR-374b expression not was significantly changed at either time point but was however associated with a subsequent development of pneumonia (p = 0.0032; Figure 1B). miR-125a3p expression was significantly reduced by 7.6% (p = 0.05) 2 hours following injury. At 24 hours, miR-125a3p was associated with the later development of infections (p = 0.015) and in particular pneumonia (p = 0.013).

## Conclusions

The expression of miRs with high predicted complementarity to the IL-10 promoter decrease following a severe traumatic injury. It is plausible that this reduction in inhibitory miRs is an important mechanism for the increase in IL-10 gene expression that is seen in these patients and thereby contributes to the consequent immunosuppressive phenotype and increased risk of nosocomial infections.
